# Barrel Shape and Chromophore Rigidity Predict Fluorescent-Protein Photophysics

**DOI:** 10.1021/acs.jcim.6c01606

**Published:** 2026-08-06

**Authors:** Luke P. Begg, Madeline L. Mason, Marc Zimmer

**Affiliations:** Chemistry Department, 5766 Connecticut College, New London, Connecticut 06320, United States

## Abstract

The 11-stranded β-barrel of fluorescent proteins (FPs) is universally conserved, yet its quantitative geometry has not been systematically characterized. We analyzed cross-sectional barrel geometry across 908 FP crystal structures in the RCSB PDB by principal component analysis (PCA)-based axis determination and convex hull analysis of protein-atom slices at the chromophore plane; 780 structures (210–245 residues, with the chromophore-containing chain selected in FP-complex cocrystals) form the canonical analysis cohort. Barrel shape, but not size, correlates with emission wavelength: red-shifted proteins have narrower, more elliptical barrels (ρ = −0.328 for minor axis, *p* = 2.2 × 10^–17^). Fluorescence quantum yield, by contrast, is not governed by barrel size: it tracks how rigidly the barrel holds the chromophore (chromophore-to-barrel B-factor ratio, ρ = −0.49 per unique FP), together with the chromophore’s ground-state planarity (ρ = −0.42) as an independent signal of comparable strength. The planarity term is most pronounced among red fluorescent proteins, which span the widest range of ground-state twist. Principal correlations survive Benjamini–Hochberg correction and partial correlation controlling for resolution. The barrel is not a passive scaffold: it constrains chromophore rigidity and thereby shapes photophysical output. The pipeline was developed with Claude (Anthropic) via Claude Code.

## Graphical abstract

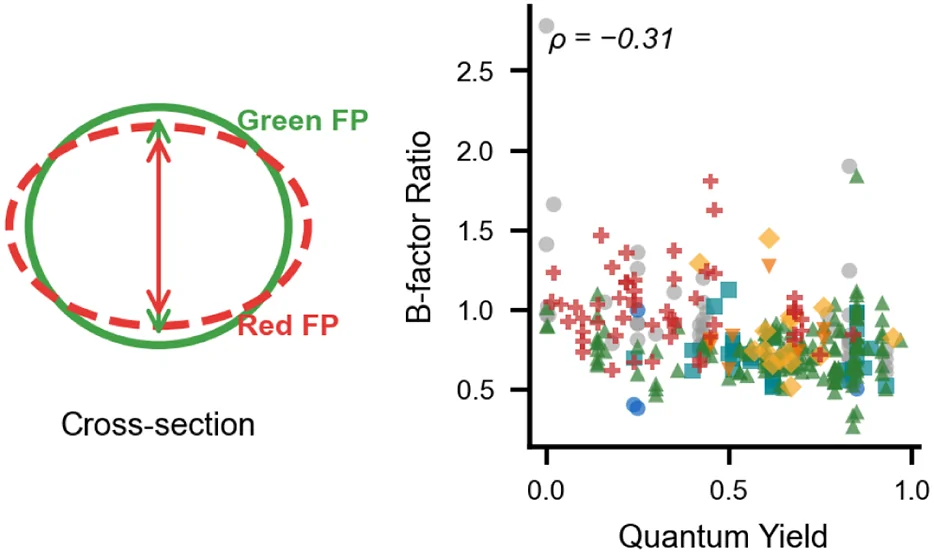

## Introduction

Green fluorescent protein (GFP), first observed in the jellyfish *Aequorea victoria* by Shimomura et al. in 1962,^1^ has fundamentally transformed biological research by enabling the direct visualization of proteins, organelles, and cellular processes in living systems.^2^ The subsequent cloning and heterologous expression of GFP by Chalfie et al.^3^ demonstrated that the protein fluoresces without requiring cofactors or external enzymes, establishing GFP as a genetically encodable marker. The significance of this discovery was recognized with the 2008 Nobel Prize in Chemistry, awarded to Shimomura^1^, Chalfie^3^, and Tsien^4^. In the decades since, fluorescent proteins have become indispensable tools across cell biology, neuroscience, and biotechnology, serving as reporters, biosensors, and partners in FRET^5^ and, more recently, super-resolution microscopy.^6,7^

The three-dimensional structure of GFP, resolved by X-ray crystallography in 1996, revealed a distinctive 11-stranded beta-barrel fold approximately 42 Å in length and 24 Å in diameter.^8,9^ The barrel surrounds a central α helix. At its center is the chromophore, which forms through autocatalytic cyclization of residues Ser65, Tyr66, and Gly67.^10,11^ The rigid encapsulation minimizes nonradiative decay, enabling high fluorescence quantum yields (the fraction of absorbed photons re-emitted as fluorescence; henceforth referred to as quantum yield). The same 11-stranded topology is conserved across all known fluorescent protein families, including GFP-derived variants and fluorescent proteins from anthozoan organisms such as DsRed, despite sequence identities as low as 25%.^12,13^

Protein engineering has produced a palette of fluorescent protein variants spanning the visible spectrum. Substitution of Tyr66 with tryptophan or histidine yields cyan or blue variants, respectively.^14^ The Thr203Tyr mutation introduces a π-stacking interaction that red-shifts emission by ∼20 nm.^15^ In the DsRed family, an additional oxidation extends the conjugated π-system, producing red emission.^16^ Structure-guided engineering has achieved notable results: Goedhart et al. obtained a quantum yield of 0.93 in mTurquoise2 through a single packing mutation (I146F) identified by structure-guided saturation mutagenesis,^17^ while Campbell and colleagues have systematically monomerized and optimized coral-derived variants.^18,19^ Hirano et al. developed StayGold, a green fluorescent protein from the jellyfish *Cytaeis uchidae*, which exhibits exceptional photostability.^20^ A persistent trend across this engineering is that quantum yield decreases with increasing emission wavelength.^21^ This has been attributed to increased vibrational degrees of freedom in extended conjugation systems, which open additional nonradiative relaxation pathways.

Although more than 800 GFP-like crystal structures are now classified in SCOP, CATH, and Pfam, the geometry of the beta-barrel itself has received little systematic study. Previous studies have focused on chromophore chemistry, hydrogen bonding networks, and site-specific mutation effects on spectral properties.^22–24^ Computational studies have shown that the barrel contracts upon chromophore maturation and that water permeability through the barrel wall varies between GFP variants.^25,26^ Analysis of conserved glycine residues at positions 31, 33, and 35 demonstrated that these residues are essential for proper barrel folding and assembly.^27^ Megley et al. showed that the φ and τ dihedral angles of the chromophore differ between yellow, blue, and green variants and correlate with nonradiative decay.^28^ Ahmed et al. used molecular dynamics to guide the engineering of YuzuFP, identifying a mutation at residue 148 that increased brightness 1.5-fold.^29^ However, the beta-barrel has generally been treated as a static scaffold. Whether barrel geometry varies systematically across fluorescent protein families, and whether such variation relates to photophysical properties, has not been addressed.

Here, we report a systematic analysis of beta-barrel cross-sectional geometry across 908 fluorescent protein crystal structures from the RCSB Protein Data Bank. For each structure, the barrel axis was determined by principal component analysis (PCA) of backbone Cα coordinates, the structure was rotated to align this axis with the *z*-axis, and a perpendicular cross-sectional slice was extracted at the chromophore level. Convex hull analysis yielded area, eccentricity, circularity, and axis lengths for each barrel. Chromophore τ and φ dihedral angles were extracted and incorporated into the analysis. These measurements were integrated with spectral data from FPbase,^30^ crystallographic B-factors, and chromophore–barrel contact counts. The results show that emission wavelength varies with barrel shape, that quantum yield depends not on barrel size but on how rigidly the barrel holds the chromophore (chromophore-to-barrel B-factor ratio, ρ = −0.49 per unique FP) and on the chromophore’s ground-state planarity (ρ = −0.42, an independent signal), and that barrel eccentricity correlates with chromophore dihedral twist.

Prior AI applications in FP research have used models trained on protein sequence or structural data for design: AlphaFold2 and RoseTTAFold to predict chromophore-related post-translational modifications,^31^ ESM3 to design the de novo FP esmGFP at 58% identity to known GFPs,^32^ and protein-specific transformers to predict fluorescence properties from sequences.^33^ In contrast, the analysis presented here uses a general-purpose conversational LLM (Claude, Anthropic; via Claude Code^34^) as a coding assistant for systematic crystallographic and statistical analysis of an existing structural database, a role following the disk-mediated multitask pattern recently described for theoretical physics by Schwartz.^35^ A short failure-mode case study (chromophore misdetection) is provided in Note S1 to illustrate the human validation required. All scientific decisions, interpretations, and responsibilities for the claims rest with the human authors.

## Methods

### Data Set Assembly

The RCSB Protein Data Bank was queried for all X-ray crystal structures belonging to the GFP-like superfamily using annotations from SCOP (b.67.1), CATH (2.60.120.200), Pfam (PF01353), and InterPro (IPR011584, IPR000786). The union returned 885 entries. Coordinate files in the mmCIF format were downloaded for all entries. Eight structures were removed after inspection (APOBEC3H complexes, a DNA cocrystal, and misannotated entries). Sequences were extracted and aligned using Clustal Omega^36^ and inspected in Jalview^37^ to confirm GFP/DsRed motifs. The initial data set comprised 877 structures; cross-referencing against additional RCSB annotations identified 31 confirmed FP barrels deposited after the last SCOP update, yielding a final data set of 908 structures (Table S1).

### Chromophore Detection

Each structure was classified by scanning nonstandard residues for known chromophore residue names. The detection list encompasses all major fluorescent chromophore variants, requiring two criteria: (i) an imidazolinone ring (atoms N2, C2, O2 in the PDB CONECT record) and (ii) a π-conjugated aromatic ring at position 66, irrespective of whether that ring carries a heteroatom donor. Phenol-based (Tyr66), indole-based (Trp66), imidazole-based (His66), and phenyl-based (Phe66) rings are all accepted; the detection logic checks for any of the standard aromatic-ring atom inventories associated with these four side chains, as well as known nonstandard naming conventions used in modified chromophore residues. Our search included Tyr66-based GFP-type chromophores (CRO, CR2, CRQ, GYC, CRG, NYG, and others), Trp66-based CFP-type chromophores (PIA, CRF, CRK, CR7, CFY, CCY, and others), His66-based BFP-type chromophores (GYS, IIC, CSH, and others), Phe66-based variants where present, and red- or orange-shifted variants (NRQ, CH6, CH7, SWG, OHD, RC7, B2H, C12, XYG, DYG, and others). In total, 71 distinct residue codes were included, among them CR8, whose aromatic ring uses nonstandard PDB atom names. Residues with an intact imidazolinone ring but no aromatic ring at position 66 (CRX, MDO, C99, CLV, KWS, Q2K, NRP, and related synthetic analogs) were excluded as nonfluorescent. A small number of early structures (e.g., 1GFL) deposit the mature chromophore as separate Ser–Tyr–Gly residues rather than as a fused CRO-type component; these were identified by the loss of the residue-65 backbone carbonyl oxygen, a signature of cyclization and dehydration, and classified as chromophore-present. Genuinely uncyclized precursor structures, which retain this oxygen, were classified as lacking a mature chromophore. Of 908 structures, 864 contained a validated mature chromophore, and 44 did not (Table S1). The chromophore detection step provides an instructive example of AI failure (Note S1).

### Chromophore Torsion Angles

Chromophore dihedral angles were defined following Megley et al.:^28^ τ = N_1_–C_1_–C_2_–C_3_ (PDB atoms N2–CA2–CB2–CG2), describing rotation about the imidazolinone–bridge bond, and φ = C_1_–C_2_–C_3_–C_4_ (atoms CA2–CB2–CG2–CD1), describing rotation about the bridge–phenol bond. Structures with |τ| < 30° were classified as *cis*, |τ| > 150° as *trans*, and the remainder as twisted. The absolute values of τ and φ are used in the correlation analyses as descriptive measures of how far the chromophore is distorted from planarity, regardless of its *cis* or *trans* configuration. The sum τ + φ is also used only as a descriptive measure of the combined rotations and is not interpreted as evidence of a hula-twist photoisomerization mechanism. Valid angles were obtained for 690 structures (Table S1).

### Barrel Axis Determination

The barrel axis was determined independently for each structure by PCA of backbone Cα coordinates. The eigenvector corresponding to the largest eigenvalue (PC1) was taken as the barrel axis. The eigenvalue ratio λ_1_/λ_2_ averaged 2.18, confirming an elongated barrel geometry. A rotation matrix mapping PC1 onto [0, 0, 1] was applied to all non-hydrogen coordinates. The cross-sectional slice was centered on the chromophore by setting the mean z-coordinate of its atoms to zero. The slice included all protein heavy atoms from standard amino acids and the chromophore with |z| ≤ 2.0 Å. See “Geometry Quantification” below for details.

### Geometry Quantification

All non-hydrogen atoms of the standard amino-acid and chromophore residues of the analyzed chain (i.e., protein heavy atoms) in each slice were projected onto the xy-plane (i.e., the plane perpendicular to the barrel axis). Ordered solvent molecules, crystallization additives, and ions were excluded from the slice so that the convex hull represents the protein barrel itself rather than the crystallographic solvent shell. The convex hull was computed using scipy.spatial.ConvexHull.^38^ Cross-sectional area, perimeter, and an ellipse fit (via the 2D covariance matrix) yielded major and minor axis lengths. Eccentricity was calculated as e = √(1 – b^2^ / a^2^). Circularity was defined as 4πA/P^2^. A ring-shape test confirmed that slices captured the barrel wall (peripheral:central density ratios of 5.4–7.0). These measurements describe the outer envelope of the beta-barrel including the side-chain wall atoms; they are therefore larger than the ∼ 24 Å barrel diameter reported for avGFP^8^ in the original crystallographic papers, which reflects the backbone framework of the barrel rather than the full side-chain envelope. The beta-sheet walls add approximately 2–3 Å on each side, consistent with the mean minor axis of 29.5 Å reported here.

### Canonical FP-Barrel Cohort

Inspection of the 908 structures showed that the largest and smallest cross-sectional areas were mainly associated with cases in which the PCA-derived barrel axis and chromophore-plane slice failed to isolate a single 11-stranded barrel. The outliers fall into two classes: (i) short constructs (<210 residues, e.g., PDB 6LOF at 163 residues) corresponding to fragments, split-GFPs, or partial barrels, and (ii) long constructs (>245 residues, e.g., GCaMP calcium sensors at ≈390 residues fused to calmodulin and the M13 peptide) in which the PC1 axis is tilted by fused domains so that the chromophore-level slice intersects nonbarrel coordinates. The monomeric FP barrel has a sequence length of approximately 220–240 residues; we therefore restricted the geometric analysis to structures with seq_length between 210 and 245 (inclusive). A secondary chain-selection audit (Table S5, Note S1b) identified 15 FP-complex cocrystals in which the original pipeline analyzed the binding partner rather than the FP chain; these entries were reprocessed using the chromophore-containing chain, after which all 15 met the canonical-cohort criteria (sequence length 210–245 residues with a mature chromophore), and 4XL5 in particular was reclassified from chromophore-absent to chromophore-positive. Separately, two entries that are not among these 15 were excluded by name because their geometry is dominated by nonstandard quaternary structure: 6H01 (domain-swapped sfGFP) and 3U0K (RCaMP, a circularly permuted red FP fused to calmodulin). The final canonical cohort comprises 780 structures. This canonical cohort eliminates major-axis outliers exceeding 50 Å while retaining all monomeric FPs identified by name-matching against FPbase. All reported barrel-geometry statistics, color-class comparisons, B-factor and dihedral correlations, and multivariate models in this paper refer to this 780-structure cohort. Section-level sample sizes (e.g., *n* = 633 for spectral correlations, *n* = 312 for the cohort-level B-factor-ratio versus quantum-yield correlation) reflect the further availability of FPbase spectral data within the canonical cohort. The full 908-structure data set, including the seq-length outliers, is retained in Table S1 and is used only for the population-level descriptive statistics in the Data set Composition subsection.

### Spectral and Photophysical Data

Spectral data were obtained from FPbase^30^ using a hierarchical four-strategy matching protocol. (1) A manually curated dictionary mapped 176 PDB entries to known FPbase proteins. (2) The FPbase GraphQL API was queried by PDB identifier for entries with registered structures. (3) PDB titles were searched against a dictionary of over 150 fluorescent protein variant names (e.g., EGFP, mCherry, Venus, Dronpa) using case-insensitive keyword matching. (4) Remaining structures were assigned spectral properties by chromophore residue type where unambiguous. When multiple strategies returned matches, the highest-priority match was retained; each PDB identifier was mapped to at most one FPbase entry. Of 908 structures, 694 (76.4%) were matched to 74 unique FPbase protein names; while 214 remained unmatched (predominantly structures with abbreviated or novel variant names not registered in FPbase). Standard FP databases often assign the wild-type avGFP quantum yield (0.79) via keyword inheritance, obscuring real within-color quantum-yield variation. We therefore recurated quantum-yield values directly from FPbase. Whenever possible, a crystal structure was matched to an FPbase entry by PDB identifier; when no direct PDB match was available, the chain-A sequence was matched to FPbase, accepting matches with at least 99% sequence identity to an entry with a curated quantum yield. This recovered quantum-yield values for 354 structures, spanning 0.0001 to 0.97, in place of uncurated annotations that contained only 14 distinct values and were dominated by repeated use of the wild-type avGFP value, 0.79. Quantum yield is a protein-level property, so replicate crystal structures of the same protein share one quantum-yield value. Quantum-yield relationships are reported at two levels: cohort-level analyses, in which each crystal structure is a data point (Figure 2A; the multivariate models), and pseudoreplication-controlled analyses, in which replicate structures are collapsed so that each protein contributes a single point (the per-unique-protein correlations in Table S6 and the robustness tests in Table S2). Extinction-coefficient values from published sources^21,30,39^ were likewise drawn from the matched FPbase entries. Color classes were assigned by emission wavelength: blue (<460 nm), cyan (460–505 nm), green (505–545 nm), yellow (545–575 nm), orange (575–610 nm), and red (>610 nm). The complete mapping is provided in Table S1.

### B-Factor and Contact Analysis

For each structure, every non-hydrogen protein atom in the analyzed chain was assigned to one of two mutually exclusive sets. The chromophore set contained all non-hydrogen atoms of the nonstandard residue identified as the chromophore, including the imidazolinone ring, the methine bridge, and the pendant aromatic ring of the Tyr66-, Trp66-, His66-, and Phe66-derived chromophores. The scaffold set contained all non-hydrogen atoms of the standard amino acid residues in the same chain. No atom belongs to both sets; water, ions, and other heteroatoms are excluded.

The scaffold set is referred to throughout this paper as the “barrel” set for brevity, but it is not restricted to the 11 β-strands of the barrel wall; it also includes the central α-helix that bears the chromophore-forming tripeptide and the capping loops at both ends of the barrel. Mean B-factors were computed for each set, and the B-factor ratio (chromophore/scaffold) quantifies chromophore rigidity relative to the rest of the chain. Chromophore–barrel contacts were counted as the number of (chromophore-atom, scaffold-atom) pairs within 4.0 Å of one another (i.e., pair count, not unique scaffold-atom count).

### Statistical Analysis

Analyses were performed in Python 3.14 using SciPy^40^ and scikit-learn.^41^ Spearman rank correlations assessed monotonic relationships between continuous variables. Mann–Whitney U tests compared two groups; Kruskal–Wallis H tests compared multiple groups. All reported p-values were corrected for multiple testing using the Benjamini–Hochberg false discovery rate (FDR) procedure. The family comprised 63 prespecified hypothesis tests covering all meaningful pairwise Spearman correlations among the six geometry metrics, four photophysical variables (em_max, lit_qy, stokes_shift, b_factor_ratio), and crystallographic resolution; 11 dihedral correlations involving τ, φ, |τ|, |φ|, the signed sum τ + φ, and ground-state planarity; plus the five Mann–Whitney chromophore-present vs absent tests, the three *cis*-vs-*trans* tests, and the three Kruskal–Wallis by color class tests. The complete list with raw and BH-corrected p-values is provided in Table S4; 45 of 63 tests survived the FDR threshold at *q* < 0.05. Partial Spearman correlations controlling for crystallographic resolution were computed by the rank-residual method: all three variables were rank-transformed, the rank-transformed dependent and independent variables were each regressed on rank-transformed resolution by ordinary least-squares, and the Pearson correlation of the residuals was reported (equivalent to the partial Spearman correlation).

### AI-Assisted Computation

All scripts were generated by Claude (Anthropic, Opus 4) via the Claude Code and reviewed by the authors before execution. Each computational task was specified in plain language; the model produced code, results were written to disk, and intermediate files were used for cross-checking against known structures. Observed failure modes (steps reported as validated without performing checks, simplification based on unrelated patterns, premature halting on the first error) were mitigated by mandatory intermediate output files and repeated verification prompts; the chromophore-detection failure is documented in Note S1. All scientific questions, methodology choices, and interpretations were made by the human authors.

### AlphaFold Structure Analysis

AlphaFold DB v6 ^42^ coordinate files were downloaded for FP sequences via the EBI AlphaFold API and matched to crystal structures via the UniProt REST API. Two analyses were performed: a paired comparison between 51 wild-type FPs for which both an AlphaFold model and the highest-resolution crystal structure exist (mean crystal resolution 1.63 ± 0.37 Å, mean pLDDT 96.7 ± 1.3); and a population-level analysis of the 309 successfully predicted AlphaFold structures whose sequence length fell within the canonical 210–245 residue window (327 successful predictions in total; 18 fell outside the window). Because AlphaFold structures lack the cyclized chromophore, the slice was centered on the barrel centroid (z = 0 in the PCA frame) rather than the chromophore z; the rest of the geometric pipeline matched the crystal-structure pipeline exactly. The pLDDT confidence scores in the AlphaFold B-factor field were not used in cross-structure comparisons with crystallographic B-factor ratios. Paired comparisons used two-sided Wilcoxon signed-rank tests; the point estimates and 95% confidence intervals reported in Results are Hodges–Lehmann pseudomedians of the paired differences and their associated distribution-free (Wilcoxon signed-rank) intervals; the Bland–Altman plots in the Supporting Information show the 95% limits of agreement (Δ̅ ± 1.96 SD).

## Results and Discussion

### Data Set Composition

The data set comprises 908 fluorescent protein crystal structures: 864 with a validated mature chromophore and 44 without. The 864 chromophore-containing structures span 70 distinct residue types (CRO, CR2, NRQ, CRQ, GYS, PIA, and others), and spectral data from FPbase were matched to 694 structures. Color classes were assigned to three additional structures based on chromophore identity or manually curated spectral information, although exact FPbase emission maxima were unavailable for these entries. Quantum yield data were available for 354. The color class distributions were green (*n* = 464), red (*n* = 95), yellow (*n* = 69), cyan (*n* = 48), orange (*n* = 11), and blue (*n* = 10).

Green fluorescent proteins, particularly avGFP-derived variants, are overrepresented in the PDB and wider literature. This imbalance probably reflects both their historical head start as the first genetically encoded fluorescent reporters and their extensive subsequent development and use. It does not necessarily indicate that green fluorescent proteins are intrinsically more useful or representative of the broader fluorescent protein family.^21^

The ten blue entries comprise nine Y66H variants identified from their His66-derived chromophore residue codes (IIC, CRG, CSH, XXY; including PDB 1BFP, 1KYP/R/S, 1EMF, 2EMD/N/O, 2FWQ) plus one Tyr-based blue variant (PDB 4ORN, ex/em 380/440 nm). The Y66H entries had been misassigned with em_max = 507 nm by FPbase keyword matching to generic “green fluorescent” titles and were reclassified directly from their chromophore residue identities. BFPs remain underrepresented in the PDB because His66-derived variants typically exhibit lower brightness and photostability than Tyr66-based counterparts,^21^ and their near-UV excitation overlaps with cellular autofluorescence; most modern applications requiring short-wavelength emission use cyan variants such as mCerulean or mTurquoise2 instead. Resolution ranged from 0.77 to 3.60 Å (mean 1.82 ± 0.44 Å). Because the PDB contains multiple entries for the same or closely related proteins, under a granular sequence-based identity (matched FPbase entry or else the chain-A sequence; Table S7), the 780 canonical structures represent 430 unique proteins. The effective sample size for cross-protein comparisons is therefore smaller than the total structure count, and robustness to pseudoreplication and sampling imbalance is assessed in Tables S2 and S7.

After applying the canonical sequence-length filter (210 ≤ residues ≤ 245) and the chain-selection audit described in Methods, the geometric analysis cohort comprises 780 structures: 742 with a validated mature chromophore and 38 without. Within this cohort, the color class distribution is green (*n* = 414), red (*n* = 90), yellow (*n* = 64), cyan (*n* = 47), orange (*n* = 11), and blue (*n* = 10), and FPbase spectral matches are available for 633 structures (318 with quantum yield data). All subsequent results refer to this canonical cohort unless explicitly stated otherwise.

### Barrel Geometry

Cross-sectional geometry was computed for the 780 structures in the canonical cohort. The mean cross-sectional area was 689 ± 40 Å^2^ (range: 566–914 Å^2^). The mean eccentricity was 0.41 ± 0.12, indicating moderately elliptical cross sections. Circularity averaged 0.92 ± 0.02. Major and minor axes averaged 32.7 ± 1.4 Å and 29.5 ± 1.3 Å, respectively. Barrel length averaged 49.3 ± 3.6 Å. Area and eccentricity were weakly positively correlated (ρ = +0.117, *p* = 0.001), reflecting the slightly larger major axis of more elliptical barrels. For comparison, we applied the same pipeline to the full data set of 908 structures, including crystallographic solvent in the slice and without auditing chain selection. This produced a much broader area distribution, with a mean of 818 ± 194 Å^2^ and a range of 330–1975 Å^2^. In the present canonical cohort, the standard deviation is approximately 4.9-fold smaller. This shows that both the cohort definition and the exclusion of crystallographic solvent remove major methodological artifacts without changing the overall shape of the distribution.

### Chromophore Maturation and Barrel Shape

Within the canonical cohort, structures with a validated mature chromophore (*n* = 742) were compared to those without (*n* = 38). Cross-sectional area did not differ (689 ± 41 vs 690 ± 29 Å^2^, *p* = 0.52), nor did circularity (0.922 ± 0.020 vs 0.919 ± 0.017, *p* = 0.24) or eccentricity (0.41 ± 0.12 vs 0.39 ± 0.11, *p* = 0.21). At the structure level, both axes were narrower in the chromophore-containing group: minor axis 29.4 ± 1.2 Å vs 30.9 ± 1.2 Å (*p* = 1.2 × 10^–10^) and major axis 32.6 ± 1.4 Å vs 33.9 ± 1.3 Å (*p* = 4.3 × 10^–9^), while the convex-hull area was preserved (Figure S1). However, the two groups in this comparison are highly unbalanced both in size (742 vs 38) and in protein diversity (208 unique chromophore-positive and eight unique chromophore-absent operational identities grouped by FPbase name where available and by PDB accession otherwise, mostly avGFP precursor variants), and the structure-level axis differences should be interpreted with this imbalance in mind. After pseudoreplication collapse to the highest-resolution structure per unique protein, the minor-axis and eccentricity differences did not survive (Table S2), whereas the major-axis difference (*p* = 8.1 × 10^–4^) and the circularity difference remained significant (*p* = 2.6 × 10^–4^). The circularity effect is small in absolute terms (0.922 vs 0.919), and both surviving differences should be read against the small, homogeneous chromophore-absent group. The paired AlphaFold–crystal comparison provides only weak, partial support for the maturation hypothesis: AlphaFold, which lacks the cyclized chromophore, yields axes shifted in the direction expected if AlphaFold barrels resembled the chromophore-absent (uncyclized) crystals, but the shifts are much smaller than the within-crystal contraction. The minor-axis shift is not significant (Δ_minor = +0.16 Å, *p* = 0.25); the major-axis shift is significant but small (Δ_major = +0.47 Å, *p* = 0.02). Both shifts are much smaller than the 1.2–1.5 Å between-group differences observed among the crystal structures. We therefore conclude only that the gross barrel cross-section is largely established at the precyclization stage and that any geometric imprint of chromophore maturation, if real, is too small to detect cleanly in this data set.

### Barrel Geometry by Emission Color Class

Barrel geometry varied with emission color class (Figure S2). Eccentricity (Kruskal–Wallis H = 113.2, *p* = 8.6 × 10^–23^), minor axis (H = 108.9, *p* = 7.0 × 10^–22^), and circularity (H = 103.0, *p* = 1.2 × 10^–20^) all differed across groups. Red fluorescent proteins had the most elliptical barrels and narrowest minor axes; green had the most circular. At the single-structure level (Figure 1), emission wavelength correlated with eccentricity (Figure 1B; ρ = +0.321, *p* = 1.4 × 10^–16^), minor axis (Figure 1A; ρ = −0.328, *p* = 2.2 × 10^–17^), and circularity (Figure 1C; ρ = −0.258, *p* = 4.5 × 10^–11^). Emission was weakly correlated with the major axis (Figure 1D; ρ = +0.176, *p* = 8.3 × 10^–6^) and uncorrelated with barrel length (ρ = −0.028, *p* = 0.47). All reported p-values were corrected for multiple testing using the Benjamini–Hochberg procedure (*q* < 0.05); the key correlations remain significant after correction. These associations also strengthen after collapsing to the highest-resolution structure per unique FPbase protein (n = 69 unique proteins): minor axis ρ = −0.54, *p* = 1.9 × 10^–6^; eccentricity ρ = +0.50, *p* = 1.5 × 10^–5^; circularity ρ = −0.47, *p* = 4.3 × 10^–5^ (Table S2), ruling out pseudoreplication of related variants as a driver. The correlations of emission with eccentricity, minor axis, and circularity also survive partial correlation analysis controlling for resolution. The major axis and barrel length are uninformative. These data are consistent with a red-shift associated with narrowing of the barrel in one dimension, rather than a general contraction. In DsRed-type red chromophores, an additional oxidation step extends the π-conjugated system by forming an acylimine group at the peptide bond N-terminal to the chromophore-forming tripeptide.^16^ The resulting larger chromophore occupies a different steric volume within the barrel pocket, which may contribute to the observed asymmetric narrowing; however, the causal direction of this relationship cannot be established from static crystal structures alone.

**Figure 1:**
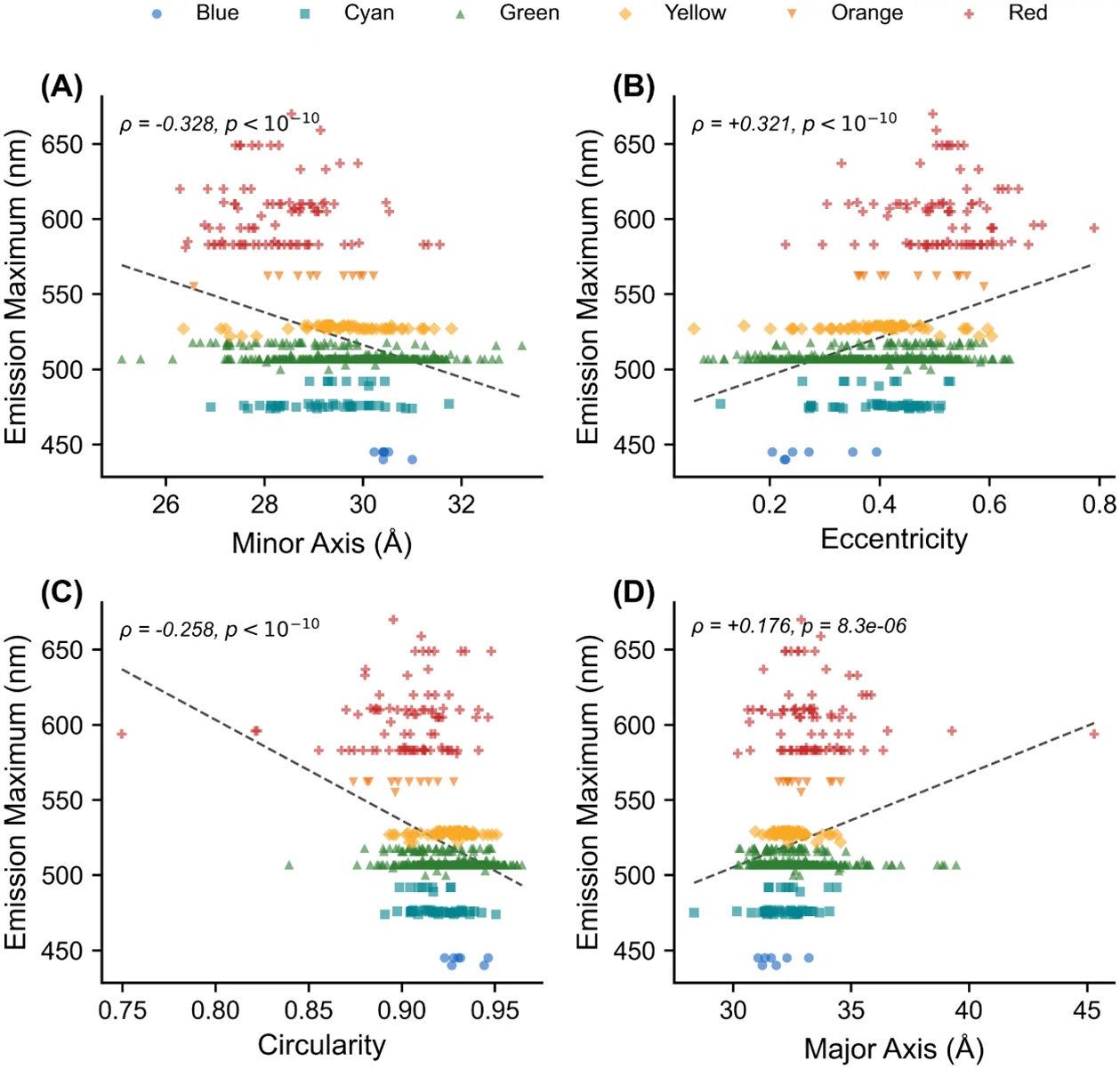
Continuous relationships between barrel geometry and emission wavelength in the canonical cohort (*n* = 633 structures with FPbase em_max). Each point represents one structure, colored by emission class. (A) Minor axis (ρ = −0.328). (B) Eccentricity (ρ = +0.321). (C) Circularity (ρ = −0.258). (D) Major axis (ρ = +0.176). The relationships are monotonic but modest, with considerable variation among individual structures. The strength of each association is therefore best assessed using the reported Spearman rank correlation coefficient and p-value, rather than by the visual spread of the data points. The dashed least-squares lines are included only as visual guides and do not imply that the relationships are linear. We also note that the associations become stronger when replicate crystal structures are combined into a single data point for each protein (Tables S2 and S7).

### B-Factor Ratio and Quantum Yield

The B-factor ratio (chromophore/barrel) was computed for 742 structures in the canonical cohort. The mean ratio was 0.81 ± 0.22; in 86.4% of structures, the chromophore was more rigid than the barrel (ratio <1). The B-factor ratio was a significant predictor of quantum yield at the cohort level (Figure 2A; ρ = −0.311, *p* = 2.1 × 10^–8^, *n* = 312), surviving partial correlation controlling for resolution (partial ρ = −0.282). When the data set is reduced to one entry per protein, this measure remains a strong and independent correlate of fluorescence quantum yield (ρ = −0.49, *n* = 123; Table S6). Its predictive strength is comparable to that of chromophore ground-state planarity, discussed below in “Chromophore Dihedral Angles.” The two measures, therefore, represent distinct structural routes to brightness and are not artifacts of replicate crystal structures. The ratio varied by color class (Figure 2C): blue 0.66 ± 0.19, cyan 0.70 ± 0.15, green 0.76 ± 0.17, yellow 0.79 ± 0.16, orange 0.88 ± 0.22, red 1.02 ± 0.24. The correlation between the B-factor ratio and quantum yield is not simply a proxy for emission wavelength. Although emission correlates with the B-factor ratio (Figure 2B; ρ = +0.421), the B-factor ratio carries information about quantum yield beyond emission wavelength alone. The physical interpretation is direct: quantum yield depends on the competition between radiative and nonradiative decay. Nonradiative decay requires conformational motion, particularly rotation about the methine bridge. A chromophore that is rigid relative to its barrel has fewer accessible conformational states and, therefore, fewer decay pathways. This interpretation is consistent with the engineering examples discussed in the Implications section below. The extended conjugation that produces red emission introduces torsional degrees of freedom that the barrel does not fully constrain, explaining the mean B-factor ratio of 1.02 ± 0.24 for red variants compared to 0.70 ± 0.15 for cyan. In red fluorescent proteins, the chromophore is, on average, no more rigid than the surrounding barrel. The green and red structures have nearly identical mean resolutions, 1.86 and 1.84 Å, respectively, but they differ in which component is better ordered.

**Figure 2:**
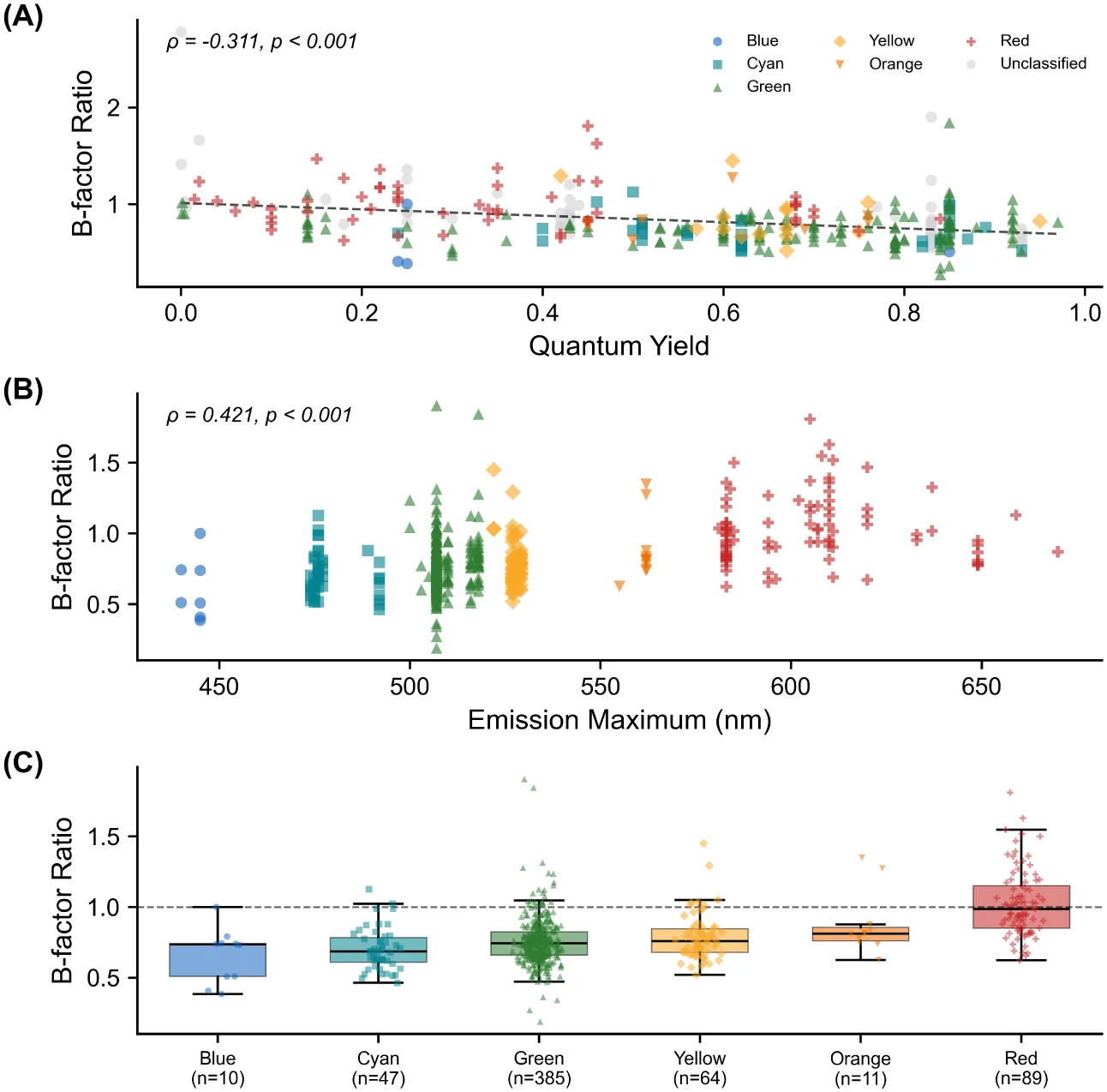
B-factor ratio analysis (canonical cohort; B-factor ratios computed for the 742 chromophore-containing structures). (A) B-factor ratio vs quantum yield (ρ = −0.311, *n* = 312). (B) B-factor ratio vs emission wavelength (ρ = +0.421, *n* = 603). (C) B-factor ratio by color class (*n* = 606): the box shows the median and IQR, with whiskers at 1.5 × IQR; individual structures are overlaid as jittered points; the dashed line at 1.0 indicates equal chromophore and barrel rigidity.

In green fluorescent proteins, the compact and deeply buried HBI chromophore is more ordered than the barrel. Its mean B-factor ratio is 0.76, with an absolute chromophore B-factor of 22.6 Å^2^ compared with 28.6 Å^2^ for the barrel. In red fluorescent proteins, the chromophore has a higher absolute B-factor of 27.7 Å^2^, which is similar to the barrel value of 26.8 Å^2^. The corresponding ratio is 1.02.

This reduced ordering is distributed across the entire red chromophore rather than being confined to the larger acylimine extension. Among 64 red fluorescent-protein structures with NRQ or CRQ chromophores, the per-atom B-factors of the acylimine backbone, the residue-1 side chain, and the conjugated core are statistically indistinguishable. Each is approximately 1.15–1.17 times the barrel Cα value.

The red chromophore, as a whole, is therefore held less rigidly relative to its barrel than the smaller green HBI chromophores. Because this conclusion is based on crystallographic B-factors, which measure atomic ordering, we do not infer a specific dynamic mechanism.

### Chromophore–Barrel Contacts

Among the 742 canonical structures containing a chromophore, the mean number of chromophore–scaffold atom pairs within 4.0 Å was 133 ± 63. This value represents the total number of atom pairs, not the number of unique scaffold atoms (see Methods). The number of contacts showed a modest positive correlation with emission wavelength (ρ = +0.203, *p* = 5.6 × 10^–7^, *n* = 600).

### Chromophore Dihedral Angles

τ and φ dihedral angles were computed for 603 canonical-cohort structures (590 of which were classifiable as *cis*, *trans*, or twisted). *Cis* configurations predominated (441, 74.7%), with 111 *trans* (18.8%) and 38 twisted (6.4%). The τ angle correlated with eccentricity (ρ = −0.197, *p* = 1.1 × 10^–6^). Absolute twist magnitudes correlated with eccentricity (|τ|: ρ = +0.167; |φ|: ρ = +0.197) and B-factor ratio (|τ|: ρ = +0.158; |φ|: ρ = +0.155). The signed sum τ + φ was the strongest dihedral predictor of emission (ρ = −0.311, *p* = 1.0 × 10^–1 2^; Figure S3C). We note that τ + φ is used here as a descriptive algebraic sum of two static crystallographic dihedral angles, not in the mechanistic sense of the hula-twist photoisomerization pathway. To relate chromophore geometry to quantum yield, we used the angular distance from the deposited (τ, φ) to the nearest planar reference, taking the minimum over the four planar settings (0°, 0°), (0°, 180°), (180°, 0°), and (180°, 180°). This folds the 180° phenol symmetry and the *cis*/*trans* degeneracy, so that a near-planar *trans* or ring-flipped chromophore is correctly scored as planar; an unfolded |τ| + |φ| sum instead misregisters such structures as maximally twisted (for example, near-planar mScarlet, φ ≈ 179°, which scores 180° rather than 2° from planar). Barrel cross-sectional measures, including area, minor-axis length, and eccentricity, show at most weak correlations with fluorescence quantum yield, with all |ρ| values below 0.16. This indicates that differences in brightness within a color class are not determined by barrel size. Instead, the relevant structural signals arise from how the barrel constrains the chromophore.

After replicate crystal structures were collapsed to one entry per protein by the granular sequence-based identity, 123 unique fluorescent proteins had both a chromophore-to-barrel B-factor ratio and a measure of ground-state chromophore planarity (Table S6). Two features showed independent correlations with fluorescence quantum yield. The chromophore-to-barrel B-factor ratio correlated with QY at ρ = −0.49 (*p* < 10^–4^), while the chromophore’s ground-state distance from planarity correlated with QY at ρ = −0.42 (*p* = 1 × 10^–4^). Each association remained after adjustment for the other, with partial correlations of ρ = −0.43 and −0.35, respectively. Neither signal is dominant, and both are modest, consistent with quantum yield being shaped by several factors, including ground-state geometry, relative chromophore mobility, and local electrostatics. The pooled per-crystal correlation based on the unfolded twist sum (ρ = −0.37) overstates the strength of this relationship. It is inflated by replicate crystal structures and by differences between color classes. It also combines the phenol-flip coordinate with true deviations from planarity.

The planarity–brightness relationship is strongest within red fluorescent proteins, which span a broad range of ground-state twist. Green fluorescent proteins, in contrast, cluster close to planar and show little variation. This pattern is consistent with differences in the chromophore torsional energy landscape contributing to the observed differences in brightness.^43^ These two structural routes are consistent with recent excited-state studies. In red fluorescent proteins, a pretwisted ground-state chromophore can lower the S_1_/S_0_ conical intersection to the Franck–Condon energy or below it, allowing nearly barrierless nonradiative decay.^44^ In engineered variants, chromophore rigidity rather than planarity alone can determine fluorescence efficiency.^45^ This finding is consistent with the independent chromophore-to-barrel B-factor-ratio signal observed here. This extends the findings of Megley et al.^28^ from a small set of structures to 603. Within this data set, τ and φ are negatively correlated across all structures (ρ = −0.357, *p* < 0.001; Figure S3A), forming two parallel bands in the τ–φ plane corresponding to *cis* and *trans* configurations. This anticorrelation primarily reflects the bimodal distribution of configurational states: *cis* structures (τ ≈ 0°) and *trans* structures (τ ≈ ± 180°) each occupy distinct, nonoverlapping regions of the τ–φ plane, such that the negative population-level correlation is a geometric consequence of the discrete classification rather than evidence for dynamic coupling between the two bonds in individual structures. Nevertheless, the relative positioning of τ and φ within each class may influence effective conjugation length. Twisted chromophores reside in more eccentric barrels with narrower minor axes. These observations are consistent with a model in which extended conjugation is associated with an asymmetric barrel that provides less symmetric constraint on torsional freedom, potentially allowing greater nonplanar distortion and increased nonradiative decay. *Trans*-configured chromophores had higher eccentricity than *cis* (0.44 vs 0.40, *p* < 0.001), lower circularity (0.918 vs 0.923, *p* = 0.019), and narrower minor axes (29.2 vs 29.6 Å, *p* = 0.003; Figure S4). The full dihedral analysis is shown in Figure S3.

In panel C, the signed sum τ + φ forms three groups. The central cluster near 0° contains *cis* chromophores, while the two outer clusters near ± 200° contain *trans* chromophores. The two *trans* clusters are separated by the sign of the wrapped dihedral angles. This pattern, therefore, reflects discrete *cis* and *trans* configurations rather than a continuous relationship with emission wavelength. Within each cluster, the red-shifted variants occur toward the top.

Panel B instead plots the symmetry-folded distance from planarity. This measure removes the *cis*/*trans* and ring-flip degeneracies. Most chromophores are close to planar and have relatively high quantum yields. A small group of strongly twisted chromophores, mainly red fluorescent proteins, consistently has lower quantum yields. This group includes the isolated point beyond 90° and produces the negative correlation between distance from planarity and fluorescence quantum yield.

### Resolution Confound and Barrel Size

If crystallographic water and other ordered solvent atoms are included in the slice, a strong negative correlation appears between resolution and cross-sectional area (ρ ≈ −0.5), because higher-resolution structures resolve more ordered water molecules, and these pad the convex hull. With the slice restricted to protein heavy atoms only (Methods), the resolution dependence of area essentially vanishes (ρ = −0.037, *p* = 0.30); the same holds for minor axis (ρ = −0.047, *p* = 0.19) and eccentricity (ρ = +0.003, *p* = 0.93). Partial correlations controlling for resolution are therefore nearly identical to the zero-order correlations: emission vs eccentricity (partial ρ = +0.322), emission vs minor axis (partial ρ = −0.328), and QY vs B-factor ratio (partial ρ = −0.282). Cross-sectional area is uncorrelated with emission (ρ = +0.039, *p* = 0.33), and barrel length is similarly uninformative (ρ = −0.027, *p* = 0.50).

Figure S5 shows the relationships between resolution and minor-axis length, as well as between emission wavelength and minor-axis length, with each point color-coded by resolution. The photophysically relevant variation appears to involve the barrel shape rather than the overall barrel size. These results suggest that the barrel shape along the minor-axis direction may be relevant to spectral tuning, whereas improving quantum yield is more likely to require direct rigidification of the chromophore.

### Stokes Shift and Barrel Geometry

The Stokes shift, which is the difference between excitation and emission maxima, was available for 633 canonical-cohort structures (mean 23.7 ± 18.6 nm, range 9–180 nm). The Stokes shift varied by color class, with rankings dominated by a small long-Stokes-shift (LSS) subset of the blue class: blue 62.9 ± 2.0 nm (*n* = 7; all LSS variants), red 40.8 ± 36.3 nm (*n* = 90, very high variance reflecting a mix of standard and large-Stokes-shift red FPs), cyan 37.9 ± 4.4 nm (*n* = 47), green 19.5 ± 9.6 nm (*n* = 414), orange 16.6 ± 8.7 nm (*n* = 11), and yellow 13.3 ± 1.3 nm (*n* = 64). The Stokes shift correlated negatively with quantum yield (ρ = −0.314, p = 1.7 × 10^–7^, *n* = 265), consistent with the interpretation that greater excited-state reorganization energy competes with radiative decay.

Stokes shift correlated significantly with barrel shape: proteins with larger Stokes shifts reside in more elliptical barrels (eccentricity: ρ = +0.127, *p* = 0.001), with narrower minor axes (minor axis: ρ = −0.132, *p* < 0.001), lower circularity (ρ = −0.142, *p* < 0.001), and shorter barrel length (ρ = −0.127, *p* = 0.001). Cross-sectional area was uninformative (ρ = −0.018, *p* = 0.66). Partial correlations controlling for emission wavelength confirmed that these associations are not simply driven by the color-class dependence of Stokes shift: eccentricity (partial ρ = +0.179), minor axis (partial ρ = −0.186), circularity (partial ρ = −0.186), and barrel length (partial ρ = −0.127) each remained significant. The barrel length association is notable given that barrel length was uninformative for emission wavelength (ρ = −0.027, *p* = 0.50), suggesting it captures variation relevant to excited-state reorganization independently of ground-state spectral tuning.

### Multivariate Analysis

To disentangle the contributions of correlated predictors, multiple linear regression was performed with quantum yield as the response variable. A model including eccentricity, minor axis, B-factor ratio, and ground-state planarity yielded *R* ^2^ = 0.18 (*n* = 250). Standardized regression coefficients show that the B-factor ratio (β = −0.257, *p* = 6.0 × 10^–5^) and chromophore ground-state planarity (β = −0.245, *p* = 6.4 × 10^–5^) are the two independently significant geometric predictors, of comparable magnitude; eccentricity (β = +0.005, *p* = 0.96) and minor axis (β = +0.074, *p* = 0.39) were not significant after controlling for the other predictors. This indicates that the bivariate correlations between barrel geometry and quantum yield are mediated through chromophore planarity and rigidity rather than barrel shape per se.

For emission wavelength, a model including minor axis, circularity, B-factor ratio, and resolution yielded *R* ^2^ = 0.33 (*n* = 600). Standardized regression coefficients again identified the B-factor ratio as the largest contributor (β = +0.394, *p* = 1.4 × 10^–25^), with circularity (β = −0.225, *p* = 6.3 × 10^–10^) and minor axis (β = −0.177, *p* = 1.8 × 10^–6^) each making smaller but independently significant contributions; resolution was marginal (β = −0.063, *p* = 0.07). Barrel shape and chromophore rigidity thus contribute to emission wavelength through partially independent pathways.

A random forest model (500 trees, bootstrap aggregation) predicting quantum yield achieved out-of-bag *R* ^2^ = 0.18, with the chromophore-to-barrel B-factor ratio as the dominant feature (permutation importance 0.53) and chromophore ground-state planarity as the second (0.41); barrel cross-sectional metrics ranked well below both.

The modest *R* ^2^ values of the linear and random forest models, both 0.18, are expected and informative rather than indicative of a deficiency. The fluorescence quantum yield depends on electronic and excited-state factors, including potential-energy-surface topology, access to conical intersections,^46^ chromophore protonation, intramolecular charge transfer, local electrostatic fields, and dark-state pathways. These properties are not captured by a static ground-state crystal structure.

A predictor based only on static structure, therefore, has a natural limit. Even with the addition of richer structural descriptors, such as hydrogen-bond counts and electrostatic terms, it produces only modest improvements. Moreover, chromophore rigidity can be more important than geometry in individual engineered variants.^45^

### Implications for Engineering

These correlations do not establish causation, and the hypotheses below remain to be tested experimentally. Nevertheless, the observed trends suggest candidate strategies. If the B-factor ratio–quantum yield correlation reflects an underlying causal relationship, then mutations that rigidify the chromophore relative to the barrel would be expected to increase quantum yield. Similarly, barrel eccentricity in a crystal structure might serve as a coarse diagnostic: high eccentricity could flag variants that are candidates for improvement, though other factors, including chromophore chemistry,^47^ protonation state, electrostatic environment,^48^ and excited-state dynamics,^46^ will also be important. The engineering successes of mTurquoise2,^17^ StayGold,^20^ and YuzuFP,^29^ each achieved through mutations altering chromophore–barrel packing, are consistent with these hypotheses but do not constitute a prospective test of them.

### AlphaFold Structural Predictions

To assess whether AlphaFold-predicted structures recapitulate crystallographic barrel geometry, a paired analysis was performed for all 51 wild-type FP sequences for which both an AlphaFold model and a matched crystal structure exist in the data set (UniProt–PDB cross-references from the UniProt REST API; highest-resolution crystal structure selected per protein; mean crystal resolution 1.63 ± 0.37 Å; mean AlphaFold pLDDT 96.7 ± 1.3). Wilcoxon signed-rank tests showed that barrel shape metrics were indistinguishable between AlphaFold and crystal structures: eccentricity (Δ = 0.00, p = 0.59) and circularity (Δ = 0.00, *p* = 0.74) did not differ. The minor axis also did not differ (Δ = +0.16 Å, *p* = 0.25, 95% CI [−0.12, +0.46]), and cross-sectional area was statistically indistinguishable (Δ = +8.4 Å^2^, *p* = 0.23, 95% CI [−5.7, +22.3]). Two metrics did show small but significant offsets: AlphaFold predicted a marginally wider major axis (Δ = +0.47 Å, *p* = 0.02, 95% CI [+0.08, + 0.86]) and a longer barrel (Δ = +1.72 Å, *p* < 10^–4^, 95% CI [+0.92, + 3.30]). Bland–Altman plots for all six metrics are shown in Figure S6. The barrel-length excess likely reflects AlphaFold modeling of flexible terminal regions that are disordered and excluded from electron density in crystal structures. The lack of a measurable cross-sectional offset is notable given that AlphaFold DB models are sequence-based and do not explicitly include the cyclized chromophore (or any post-translational modification) in the predicted structure: the chromophore-forming residues are output as the uncyclized Ser–Tyr–Gly precursor. The barrel cross-section is therefore reproduced from sequence information alone.

Second, a population-level comparison was performed between the 309 AlphaFold structures and the 742 chromophore-containing crystal structures in the canonical cohort. With crystallographic solvent excluded from the crystal slice, the size of the AlphaFold and crystal barrels is similar: the AlphaFold area is only 11 Å^2^ larger than the crystal mean (700 ± 49 vs 689 ± 41 Å^2^, p = 1.5 × 10^–4^), but the shape of the predicted barrel differs systematically. AlphaFold barrels have a narrower minor axis (28.7 ± 1.3 vs 29.4 ± 1.2 Å, Δ = −0.7 Å, *p* < 10^–15^), a wider major axis (33.8 ± 1.6 vs 32.6 ± 1.4 Å, Δ = +1.1 Å, *p* < 10^–28^), higher eccentricity (0.51 ± 0.11 vs 0.41 ± 0.12, *p* < 10^–32^), and lower circularity (0.91 ± 0.02 vs 0.92 ± 0.02, *p* < 10^–32^). These population differences partly reflect the distinct protein compositions of the two data sets: the AlphaFold set includes many uncrystallized FPs that may be enriched in red-shifted, more eccentric variants, while the crystal set is dominated by well-studied green FPs. Notably, the correlation between emission maximum and minor axis length prominent in crystal structures (ρ = −0.33, *p* < 10^–16^) was absent in the AlphaFold ensemble (ρ = −0.018, *p* = 0.85). Instead, sequence length remained a structural correlate even within the canonical filter window (ρ = −0.42 with eccentricity, *p* < 0.0001). This pattern is consistent with the mature chromophore contributing to the barrel distortions associated with emission wavelength, although differences in data set composition prevent a causal conclusion. AlphaFold therefore provides a useful geometric baseline for uncrystallized FPs, but reproducing the spectral correlations requires experimentally determined or computationally modeled chromophore geometry.

### Limitations

The canonical cohort is unevenly distributed (green *n* = 414; blue and orange ≤11), and the PDB itself is not a random sample of all FPs; heavily studied variants such as GFP and mCherry are overrepresented, while many natural and engineered FPs have no deposited structure. We addressed the sampling imbalance and pseudoreplication directly (Table S7). Using a granular sequence-based identity (430 unique proteins in the cohort, assigned by matched FPbase entry or else the chain-A sequence rather than by generic name), the principal correlations are preserved or strengthened when replicate crystals are collapsed to one entry per protein; redundancy attenuated rather than inflated them. They also hold in a monomer-only rerun (emission vs minor axis ρ = −0.46; QY vs B-factor ratio ρ = −0.51), ruling out oligomeric-packing artifacts, and the emission–geometry relationship holds within the green class alone (minor axis ρ = −0.20, *p* = 3 × 10^–5^). The blue (*n* = 10) and orange (*n* = 11) classes are too small to support reliable within-class analysis and are therefore described only qualitatively. Quantitative within-class conclusions are limited to the better-sampled green, red, cyan, and yellow classes. The broader family-level correlations remain robust in the monomeric subset, but their generalizability to fluorescent proteins without available crystal structures remains uncertain.

Crystal packing is another limitation. Contacts within the crystal lattice can impose nonphysiological forces and alter barrel eccentricity, particularly for proteins crystallized in different space groups.

Crystallographic B-factors reflect several factors in addition to true molecular mobility, including resolution, refinement protocol, occupancy, TLS treatment, and crystal packing. Our B-factor ratio reduces these effects by normalizing the chromophore to the surrounding protein within each structure. The relationship also remains after controlling for resolution, with a partial correlation of ρ = −0.282. Even so, the ratio should be interpreted as a proxy for relative chromophore mobility rather than as a direct measure of dynamics.

The result is also insensitive to the choice of reference atoms. Replacing the whole-chain scaffold with the atoms in the chromophore-plane cross-section, defined by the convex-hull slice, produces almost no change in either the B-factor ratio or its correlation with fluorescence quantum yield. The Spearman correlation changes by less than 0.01, and the two versions of the ratio correlate at ρ = 0.94. Thus, the result does not depend on including the capping loops or central helix in the reference set.

The geometry pipeline is similarly robust. Sensitivity tests using slice thicknesses from 1.5 to 2.5 Å and either all protein heavy atoms or backbone atoms alone preserved the rank ordering of structures by eccentricity, circularity, and minor-axis length (Table S3).

Fluorescence quantum yield values were available for only 318 structures in the canonical cohort and were measured under heterogeneous experimental conditions. A Spearman correlation matrix for selected structural, photophysical, and crystallographic variables is provided in Figure S7, and barrel geometry grouped by chromophore type is shown in Figure S8.

## Conclusions

We analyzed a canonical cohort of 780 fluorescent protein crystal structures selected from 908 total deposits. Across this cohort, barrel shape, rather than overall size, was associated with emission wavelength. Red-shifted variants had narrower and more elliptical barrels, whereas cross-sectional area and barrel length showed little relationship with emission.

Fluorescence quantum yield was also only weakly related to barrel size. Stronger structural signals came from how the barrel restrained the chromophore. The chromophore-to-barrel B-factor ratio was the strongest barrel-relative correlate of quantum yield, with ρ = −0.49 when calculated per unique fluorescent protein. Ground-state chromophore planarity provided an independent signal of similar strength, with ρ = −0.42, and this relationship was most pronounced among red fluorescent proteins.

Barrel eccentricity was also correlated to chromophore dihedral twist. This extended the observations of Megley et al.^28^ from a small number of structures to 603 structures in the canonical cohort.

AlphaFold reproduced both the size and shape of the fluorescent protein barrel. In paired comparisons with high-resolution crystal structures, only the major axis and barrel length differed measurably, by +0.5 Å and +1.7 Å, respectively. The minor axis, cross-sectional area, eccentricity, and circularity were statistically indistinguishable from the crystallographic values.

Together, these results show that the barrel is not simply a passive scaffold. Its geometry is associated with the emission wavelength, and the extent to which it restrains the chromophore is associated with the fluorescence quantum yield. The chromophore-to-barrel B-factor ratio may, therefore, provide a useful structural diagnostic for identifying variants that could benefit from improved chromophore rigidification. This may be particularly valuable for red-shifted fluorescent proteins, in which the chromophore is, on average, no more rigid than the surrounding barrel.

The structural patterns identified here motivate prospective experimental work, particularly mutations targeting the minor-axis-defining strands of red FPs, aimed at restoring the rigidity differential that characterizes brighter green and cyan variants. On the methodological side, the entire pipeline was generated with AI assistance (Claude Code); the chromophore-detection failure documented in Note S1 illustrates that intermediate output files and explicit cross-checks against known structures remain essential when LLMs are used as coding agents for scientific data.

## Supplementary Material





## Data Availability

All Python scripts used for CIF parsing, PCA-based barrel axis determination, cross-sectional slicing, convex hull analysis, spectral data matching, B-factor extraction, dihedral angle computation, statistical analysis, and figure generation were developed with the assistance of Claude (Anthropic) via Claude Code and are available at https://github.com/headsparkle/gfp-barrel-geometry. The complete per-structure data set (Table S1) is provided as a CSV file in the Supporting Information.

## Notes

Barrel shape and chromophore rigidity predict fluorescent-protein photophysics (PDF)
Data used for all computational analyses, statistical processing, and generation of the figures and supplementary tables presented in this study­(ZIP)
