# Supplementary material for: Barrel Shape and Chromophore Rigidity Predict Fluorescent-Protein Photophysics
Source: J Chem Inf Model. 2026 Aug 6;66(16):10266–76. doi: 10.1021/acs.jcim.6c01606 (PMC13508766; doi:10.1021/acs.jcim.6c01606)
Supplement: Supplementary file 1 [file ci6c01606_si_001.pdf]

Supporting Information

Barrel Shape and Chromophore Rigidity Predict Fluorescent-Protein Photophysics

Luke P. Begg, Madeline L. Mason, Marc Zimmer*

Chemistry Department, Connecticut College, New London, CT 06320, United States

*E-mail: mzim@conncoll.edu

The Supporting Information contains the following items:

Table S1. Full analysis dataset of 908 fluorescent-protein structures with PDB identifiers, chromophore types, cross-sectional geometry parameters, spectral data, B-factor ratios, dihedral angles, chromophore–barrel contact counts, photophysical properties, and a Canonical_Cohort flag identifying the 780 structures (sequence length 210–245 residues, with two structural-topology outliers excluded; see Note S1(b)) that comprise the geometric-analysis cohort reported in the main text. This table also serves as the source data for all figures in the manuscript (provided as a CSV file). PDB entries 4DXQ and 4DXP (Kaede photoconvertible FP) are genuine FP barrels present in the PDB but absent from this dataset because their SCOP classification was not captured in our curation pipeline; they were not explicitly excluded on scientific grounds.

Table S2. Pseudoreplication robustness test. Key correlations and group comparisons recomputed after collapsing to one structure per unique protein within the canonical cohort. Two collapse rules are used: (i) for Spearman correlations and Kruskal–Wallis tests, which rely on FPbase spectral data, the 633 canonical-cohort entries with em_max are collapsed by unique FPbase match name (n = 69 unique proteins, highest-resolution entry retained per protein); (ii) for the three Mann–Whitney tests of chromophore-present vs chromophore-absent barrels, the

full canonical cohort is collapsed by FPbase match name where available, falling back to PDB id otherwise (n = 216; 208 chromophore-positive, 8 chromophore-absent). The "Original" columns are the canonical-cohort values reported in the main text; the "Unique" columns are the values after collapse. The Survives column flags whether each finding remained significant at $p < 0.05$; ten of twelve survive. The two that do not survive are the eccentricity and minor-axis Mann–Whitney tests of chromophore-present vs chromophore-absent barrels, reflecting the small number of unique chromophore-absent proteins (n = 8) (provided as a CSV file).

Table S3. Sensitivity analysis of the geometry pipeline. Cross-sectional parameters were recomputed for 52 representative structures using slice thicknesses of 1.5 Å and 2.5 Å (vs the default 2.0 Å) and using backbone-heavy atoms only (vs the default protein heavy-atom selection). Pearson and Spearman rank correlations with the default parameterization are reported (provided as a CSV file); the rank order of structures by eccentricity, circularity, and minor axis is preserved across all variants.

Table S4. Benjamini–Hochberg multiple-testing correction for the full family of 63 pre-specified hypothesis tests on the canonical cohort. Each row lists the test name, test type (Spearman, Mann–Whitney, or Kruskal–Wallis), the test statistic (ρ, U, or H), the sample size, the raw p-value, the BH-adjusted p-value, and whether the test survives the FDR threshold $q < 0.05$ (provided as a CSV file). 45 of 63 tests survive correction; the eighteen that do not are predominantly low-effect-size correlations involving cross-sectional area, crystallographic resolution, and barrel length, none of which is interpreted as a key finding in the main text.

Table S5. Chain-selection audit output (per-PDB). For each of the 908 PDB entries, the audit script enumerates every polymer chain, identifies which chain(s) contain a recognized

mature chromophore residue, records the chain the original geometric pipeline would have used (the longest standard-amino-acid chain), and flags entries where the pipeline analyzed a non-chromophore chain when another chain in the same entry contains the chromophore (`bug_flag = True`). See Note S1(b) for the discussion of the 15 flagged entries and the action taken (provided as a CSV file).

Table S6. Per-unique-FP quantum-yield correlations for the two independent ground-state structural predictors, computed with replicate crystals collapsed to one entry per protein by median (n = 123 unique FPs with both metrics, no spectral gate). Ground-state planarity is the symmetry-folded distance from the deposited (τ, φ) to the nearest planar reference; chromophore planarity (ρ = –0.42) and the chromophore/barrel B-factor ratio (ρ = –0.49) each survive adjustment for the other (partial ρ = –0.35 and –0.43), confirming two distinct, independent structural routes to quantum yield (provided as a CSV file).

Table S7. Sampling and pseudoreplication robustness. Each key correlation is re-evaluated under several subsampling regimes: the full cohort (per structure), per unique FP (granular protein identity), monomer-only (per unique FP; oligomeric state from FPbase), excluding the two smallest classes (blue + orange), and within the green class alone. The correlations are preserved or strengthened under per-protein collapse and in the monomer-only subset, and the emission–geometry relationship holds within green alone, indicating the findings are not artifacts of oligomeric packing, redundant crystal entries, or green-class dominance (provided as a CSV file).

Note S1. AI Failure Case Studies. Three episodes during this work illustrate characteristic ways that LLM-generated code can be confidently wrong, and the kinds of human expert checks that catch them.

(a) Chromophore detection. The initial implementation used a hardcoded list of 31 residue names to identify mature chromophores. This list was incomplete in two directions. First, approximately 40 additional chromophore residue codes present in the dataset were omitted, causing approximately 90 structures to be incorrectly classified as lacking a chromophore; among these were 1BFP (BFP-type chromophore IIC, His66-derived), 1EMF and 1EMK (CFP-type variants CSH and CCY, Trp66-derived), and others. Second, the residue code CR8 was present in the original list on the basis of its name resemblance to other chromophore codes (CR2, CR7, CRO), but the atom inventory check used standard Tyr66 atom names (CG2, CD1, CD2, CE1, CE2, CZ, OH) that are absent from CR8; CR8 instead uses a nonstandard naming convention (C4–C8, C11, C12, O13 for the phenol ring), so the aromatic ring was missed and 21 CR8-containing structures were incorrectly reclassified as non-chromophoric. Examination of the structures confirmed that CR8 contains both a complete imidazolinone ring and a para-hydroxyphenyl group bridged through the methine carbon (C8=CA2), consistent with a Tyr66-derived chromophore; the Arg residue at sequence position 66 in these entries corresponds to the conserved Arg96 in biological GFP numbering, which participates in chromophore formation but is not part of the chromophore itself. This class of error, confidently wrong results from incomplete pattern matching, was caught only by inspecting per-entry intermediate output against known structures.

(b) Chain selection in FP-complex co-crystals. The default chain-selection rule analyzed the longest standard-amino-acid chain in each PDB entry. For 15 entries this is the wrong chain:

the FP is co-crystallized with a longer binding partner (designed ankyrin / armadillo repeat protein, antibody, or nanobody fragment), and the binder was therefore analyzed in place of the FP. The flagged entries are 4XL5, 5AQB, 5LEL, 5LEM, 5MA3, 5MA4, 5MA5, 5MA9, 5MAK, 5MFC, 8BAN, 8BAV, 8RZZ, 8S0G, and 8S1L. A chain-selection audit (`scripts/audit_chain_selection.py`, full output in Table S5) parses every deposited mmCIF, lists all polymer chains, and flags entries where the longest chain does not contain a chromophore but another chain in the same entry does. After reprocessing each flagged entry with the chromophore-containing chain, all 15 yielded barrel geometry consistent with the canonical cohort. A separate outlier scan identified two further entries whose geometry is dominated by nonstandard quaternary structure rather than a monomeric β-can: 6H01 (a domain-swapped dark-state sfGFP carrying an ONBY-66 caged chromophore; major axis 35.6 Å vs the cohort mean of 32.7 Å, eccentricity 0.65 vs the mean 0.41, because the β-strands cross between monomers) and 3U0K (RCaMP, a circularly permuted red FP fused to calmodulin and an M13 peptide for calcium sensing; major axis 51.3 Å, eccentricity 0.87, reflecting the cpFP topology). Both are well-characterized FP-derived proteins but do not represent a standard monomeric FP barrel, and are excluded from the canonical cohort by name.

(c) Crystallographic solvent inside the chromophore-plane slice. Every version of the pipeline preceding the present submission constructed the cross-sectional convex hull from all atoms within ±2.0 Å of the chromophore plane, including ordered water molecules and ions. This inflated the mean cross-sectional area by roughly 10% and, more damagingly, produced a strong but spurious correlation between crystal resolution and area ($\rho \approx -0.49$): high-resolution structures simply contained more visible water inside the barrel, so the apparent "wider barrel at high resolution" was a solvent-modeling artifact rather than a structural feature. The issue first

became visible in 5AQB, which had a band of internal waters running through the chromophore plane and a fitted area far above its cohort neighbors. Restricting the slice to protein heavy atoms (standard amino-acid residues plus the chromophore residue) eliminated the artifact: mean area dropped from 776 to 689 Å^2, the resolution–area Spearman correlation collapsed from –0.49 to –0.04, and 5AQB returned to a normal-looking barrel without needing to be excluded. The 776-to-689 Å^2 comparison here is within the canonical cohort; the 818 Å^2 mean quoted in the main text is the corresponding value for the unaudited full 908-structure dataset. All numbers reported in this manuscript use the protein-only slice. The episode is a reminder that a strong correlation produced by an LLM-generated pipeline can be entirely an artifact of an unstated atom-selection choice.

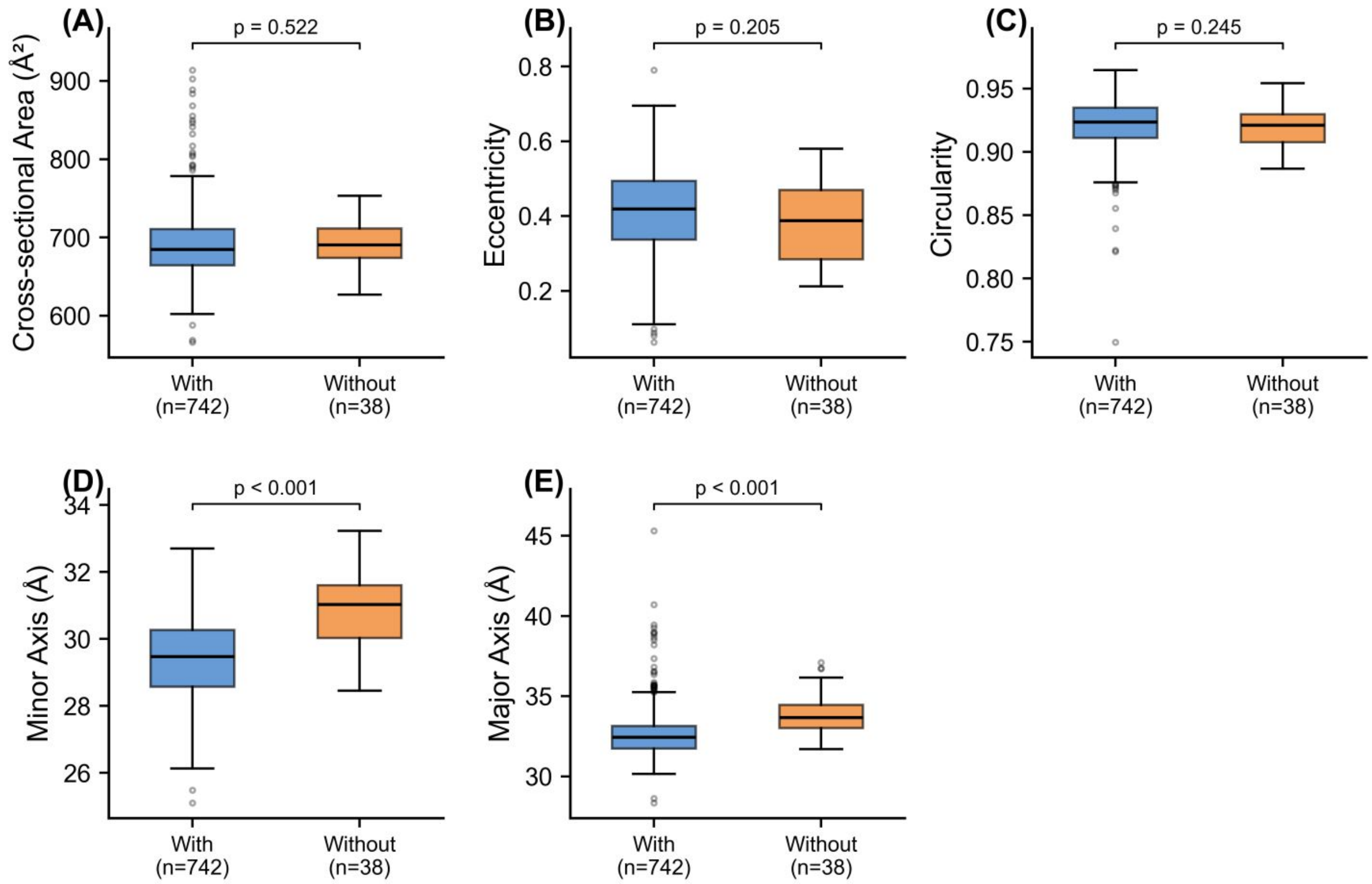

Figure S1. Effect of chromophore maturation on barrel geometry within the canonical cohort (n = 780). Boxplots compare structures with (n = 742, blue) and without (n = 38, orange) a validated mature chromophore. (A) Cross-sectional area, (B) eccentricity, and (C) circularity do not differ between the two groups. (D) Minor axis and (E) major axis are both narrower in chromophore-containing structures at the structure level ($p < 10^{-7}$ for both); the minor-axis difference does not survive pseudoreplication collapse to one highest-resolution structure per unique protein, whereas the major-axis difference remains significant ($p = 8.1 \times 10^{-4}$; Table S2); both should be interpreted against the small number of unique chromophore-absent proteins (n = 8) and the unbalanced group sizes. See main-text Chromophore Maturation section for interpretation.

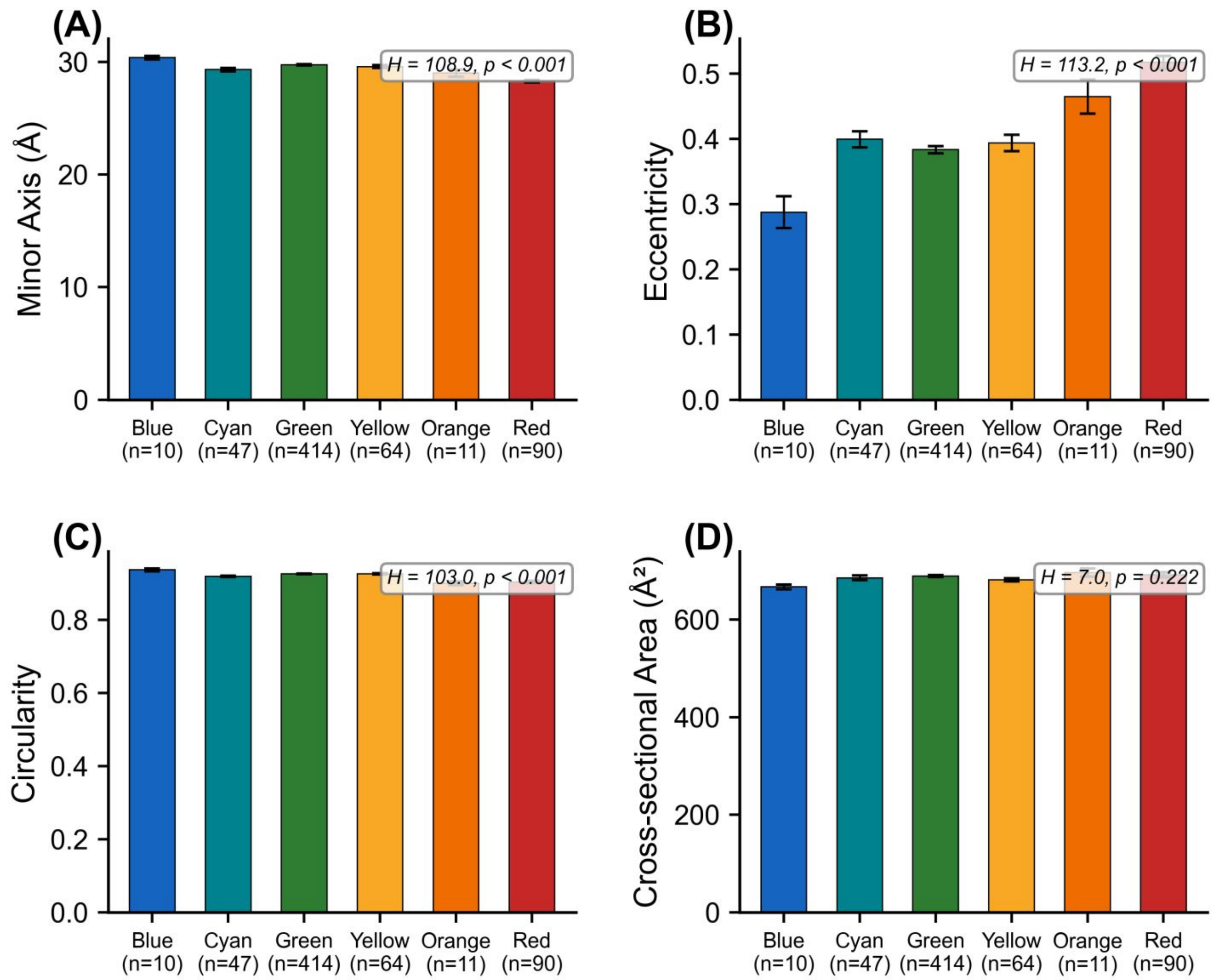

Figure S2. Barrel geometry by emission color class within the canonical cohort. Mean (± SEM) minor axis, eccentricity, circularity, and cross-sectional area by color class, with Kruskal–Wallis H and p-values annotated. This is the categorical summary of the continuous relationships shown in Figure 1.

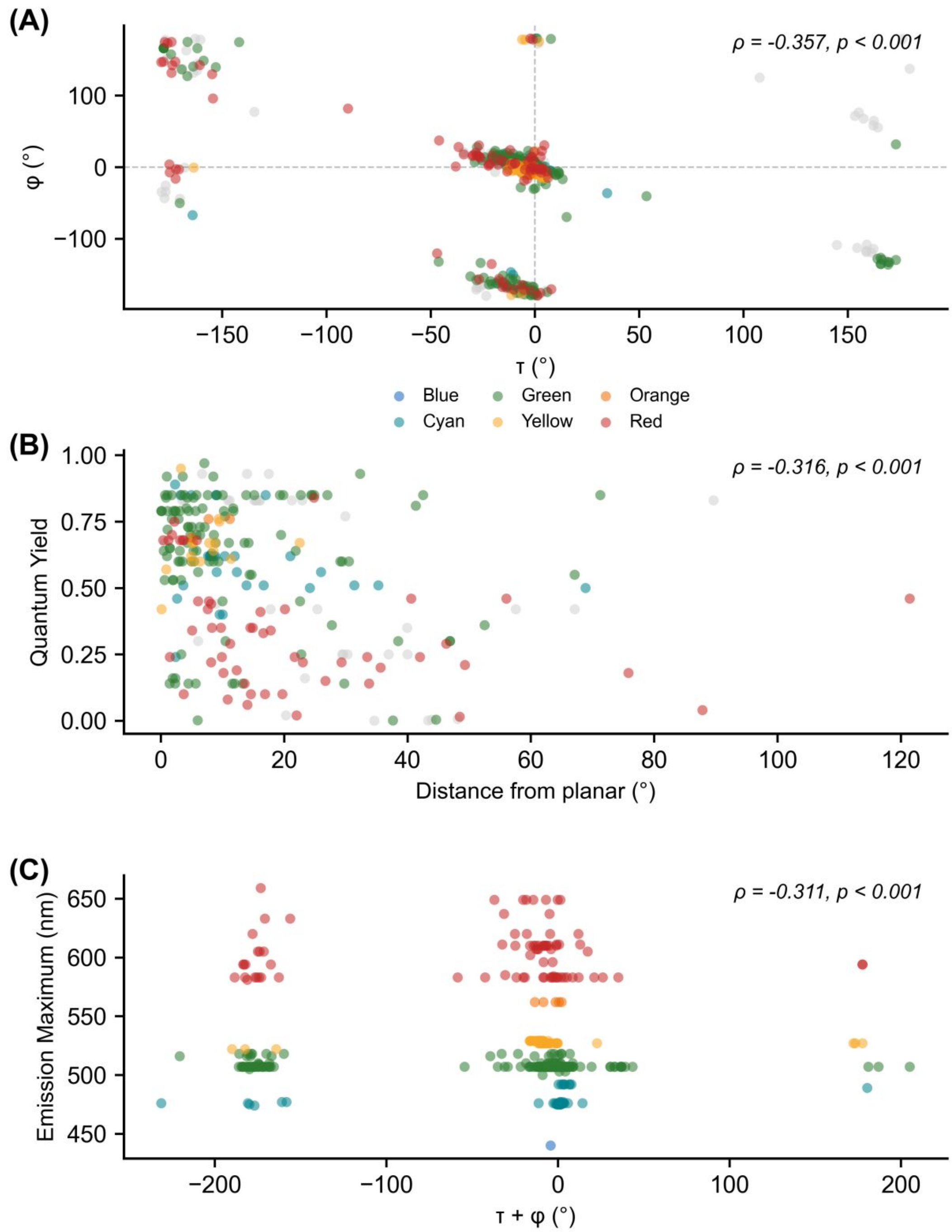

Figure S3. Chromophore dihedral analysis. (A) τ vs φ plot colored by emission class. (B) Ground-state planarity (distance from the nearest planar reference, folding the phenol symmetry) vs quantum yield; the panel shows all canonical structures (per-structure ρ = –0.32), while the per-unique-FP value is ρ = –0.42 (Table S6). (C) Dihedral sum (τ + φ) vs emission wavelength.

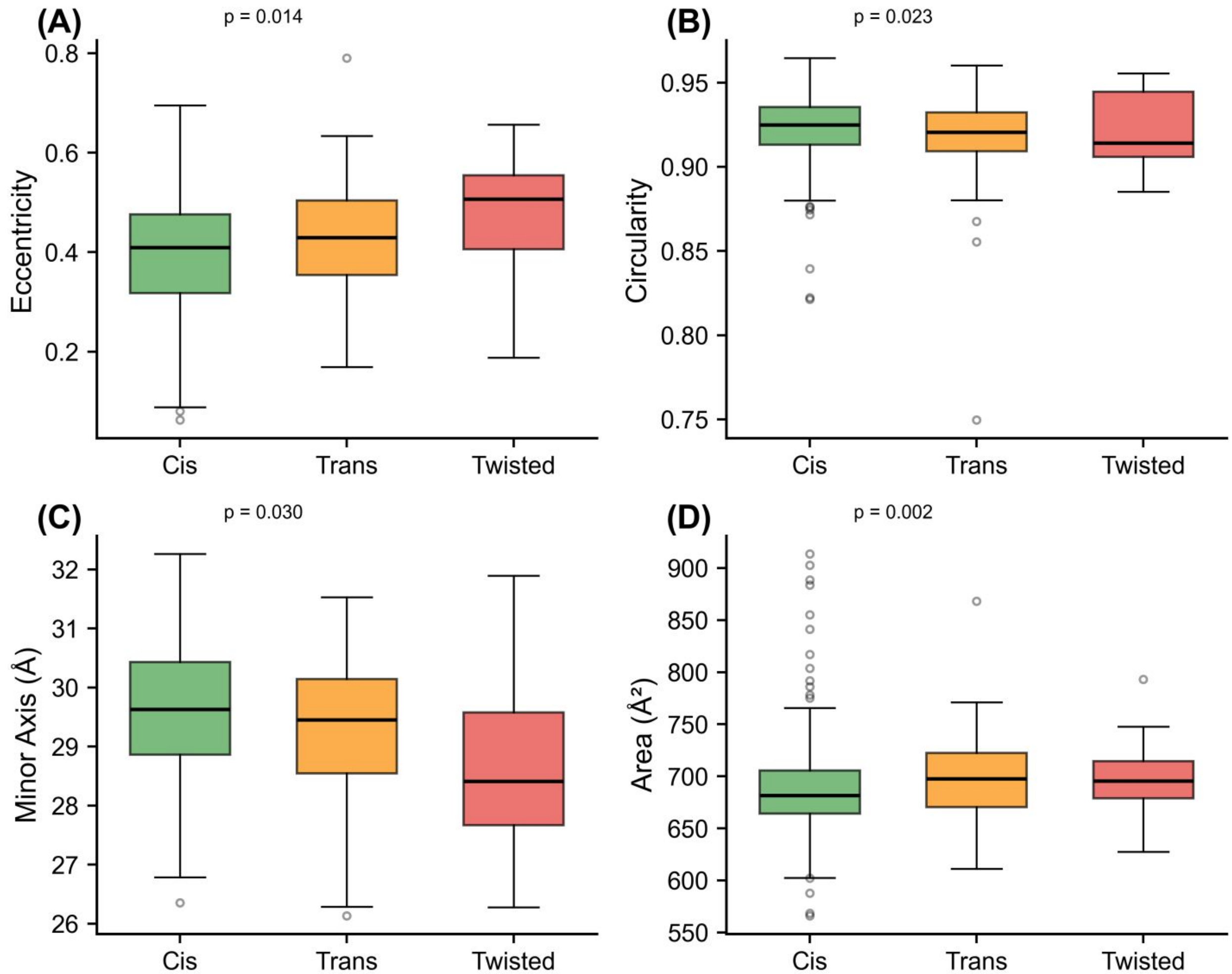

Figure S4. Barrel geometry by chromophore configuration (*cis*, *trans*, and twisted). Displayed p-values are two-sided Mann–Whitney tests comparing the *cis* and *trans* groups; the twisted group is shown for context but is not part of the plotted comparison.

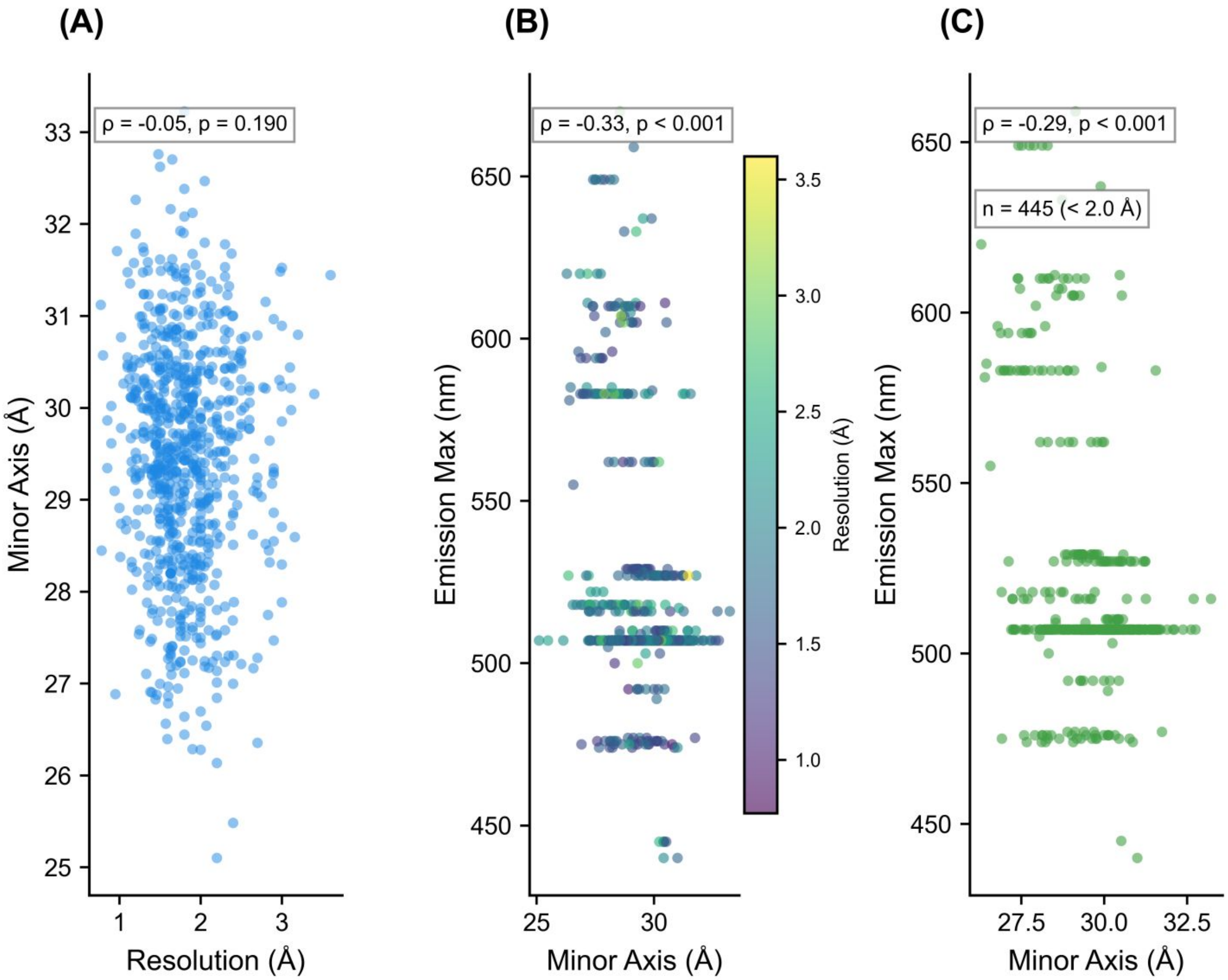

Figure S5. Resolution control. (A) Resolution vs minor axis. (B) Emission vs minor axis colored by resolution. (C) High-resolution subset (<2.0 Å) only.

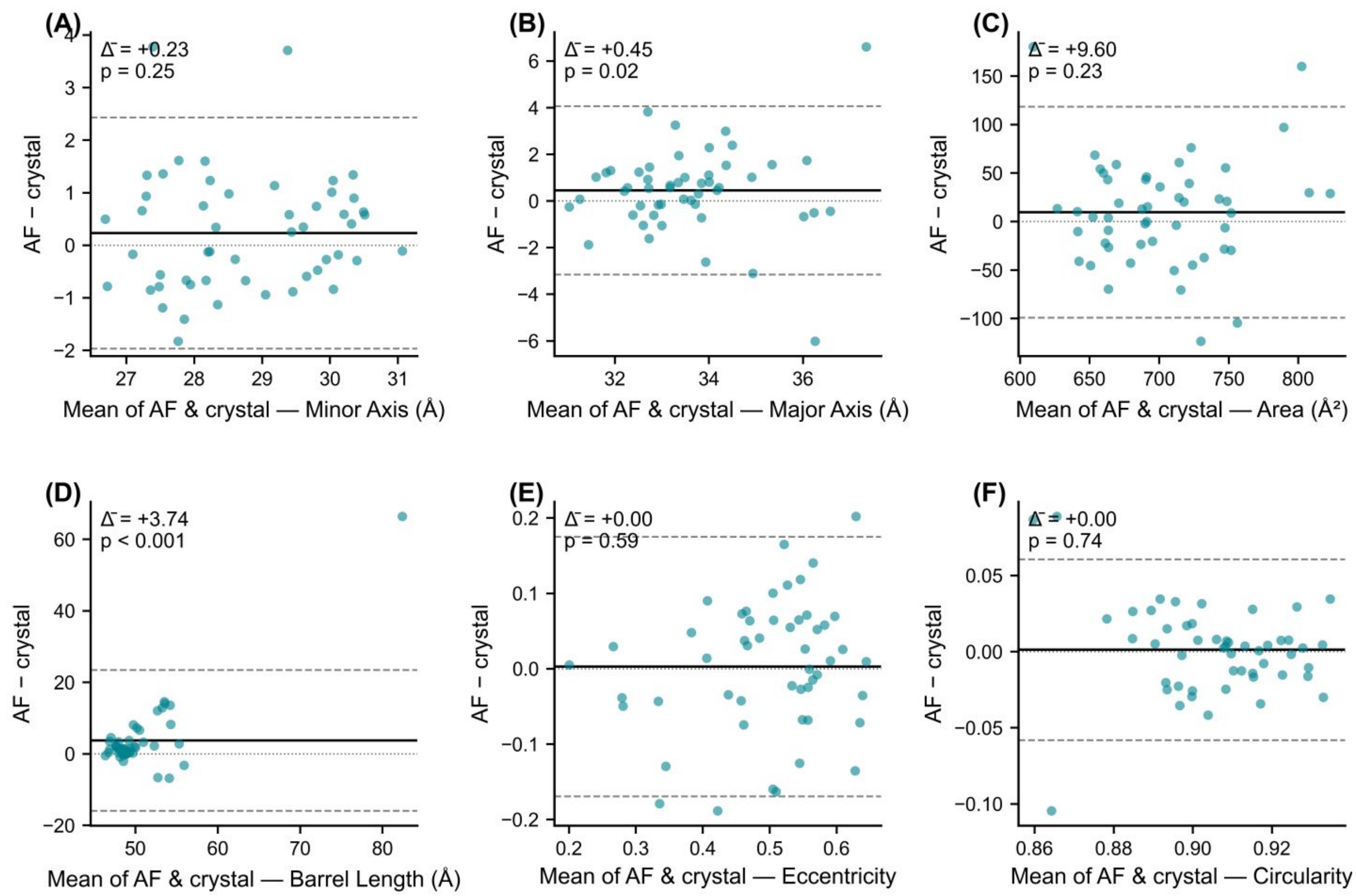

Figure S6. Bland–Altman plots of the paired AlphaFold–crystal comparison ($n = 51$ wild-type FPs, one paired observation per protein). Each point is one protein; the x-axis is the mean of the AlphaFold and crystal values and the y-axis is AlphaFold minus crystal. The solid horizontal line marks the mean difference ($\bar{\Delta}$); dashed lines mark the 95% limits of agreement ($\bar{\Delta} \pm 1.96$ SD); the dotted line at zero marks perfect agreement. p-values are from two-sided Wilcoxon signed-rank tests. With crystal slices restricted to protein heavy atoms (no ordered solvent), AlphaFold and crystal barrels are statistically indistinguishable on minor axis ($\bar{\Delta} = +0.23$ Å, $p = 0.25$), eccentricity ($\bar{\Delta} = 0.00$, $p = 0.59$), circularity ($\bar{\Delta} = 0.00$, $p = 0.74$), and area ($\bar{\Delta} = +9.6$ Å², $p = 0.23$). AlphaFold predicts slightly wider major axes ($\bar{\Delta} = +0.45$ Å, $p = 0.02$) and longer barrels ($\bar{\Delta} = +3.7$ Å, $p < 10^{-4}$). The mean differences shown in the Bland–Altman plots differ slightly from the Hodges–Lehmann pseudomedians reported in the main text because the two analyses use different estimators.

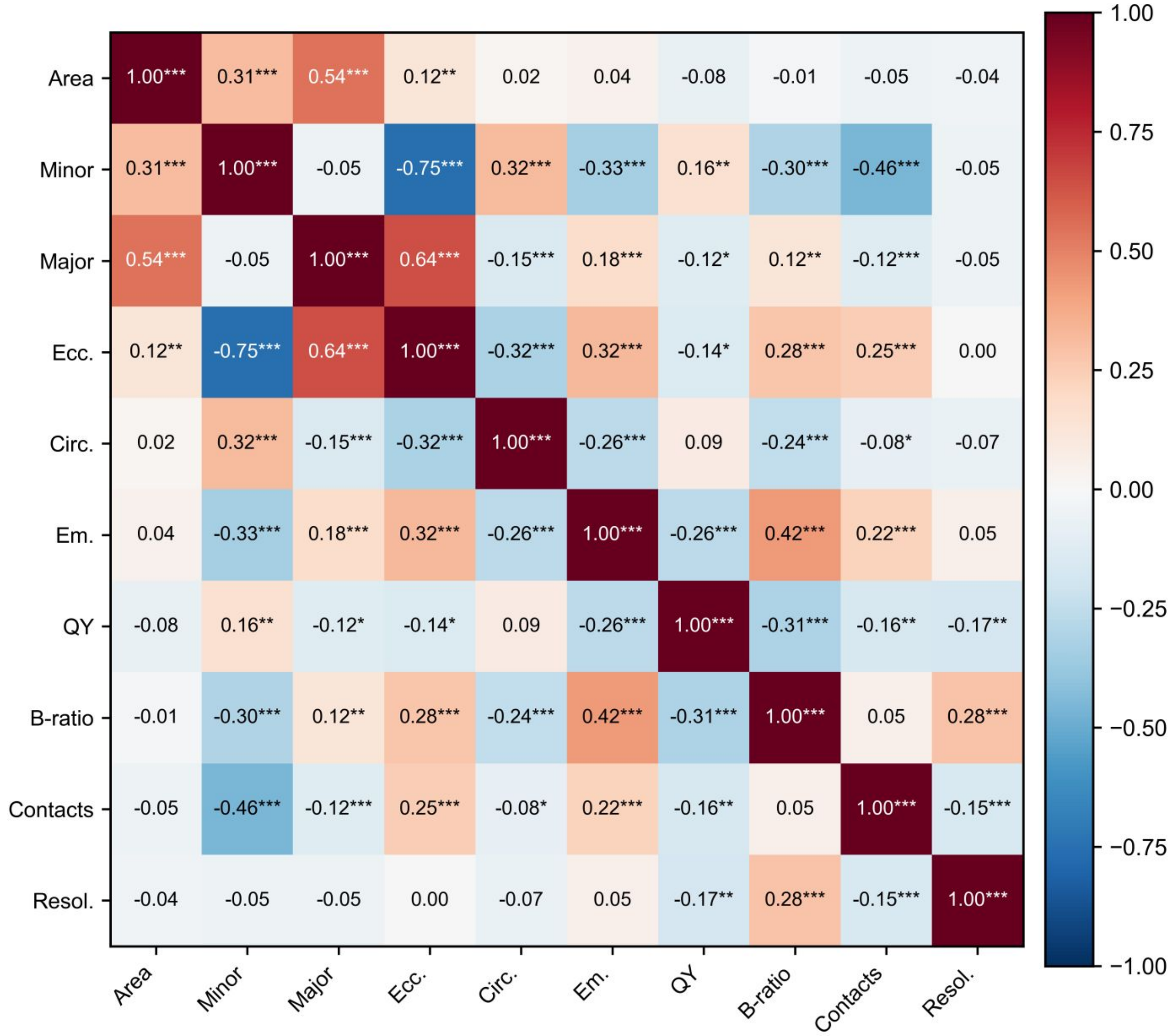

Figure S7. Spearman correlation matrix for selected structural, photophysical, and crystallographic variables. Asterisks indicate * $p < 0.05$, ** $p < 0.01$, *** $p < 0.001$.

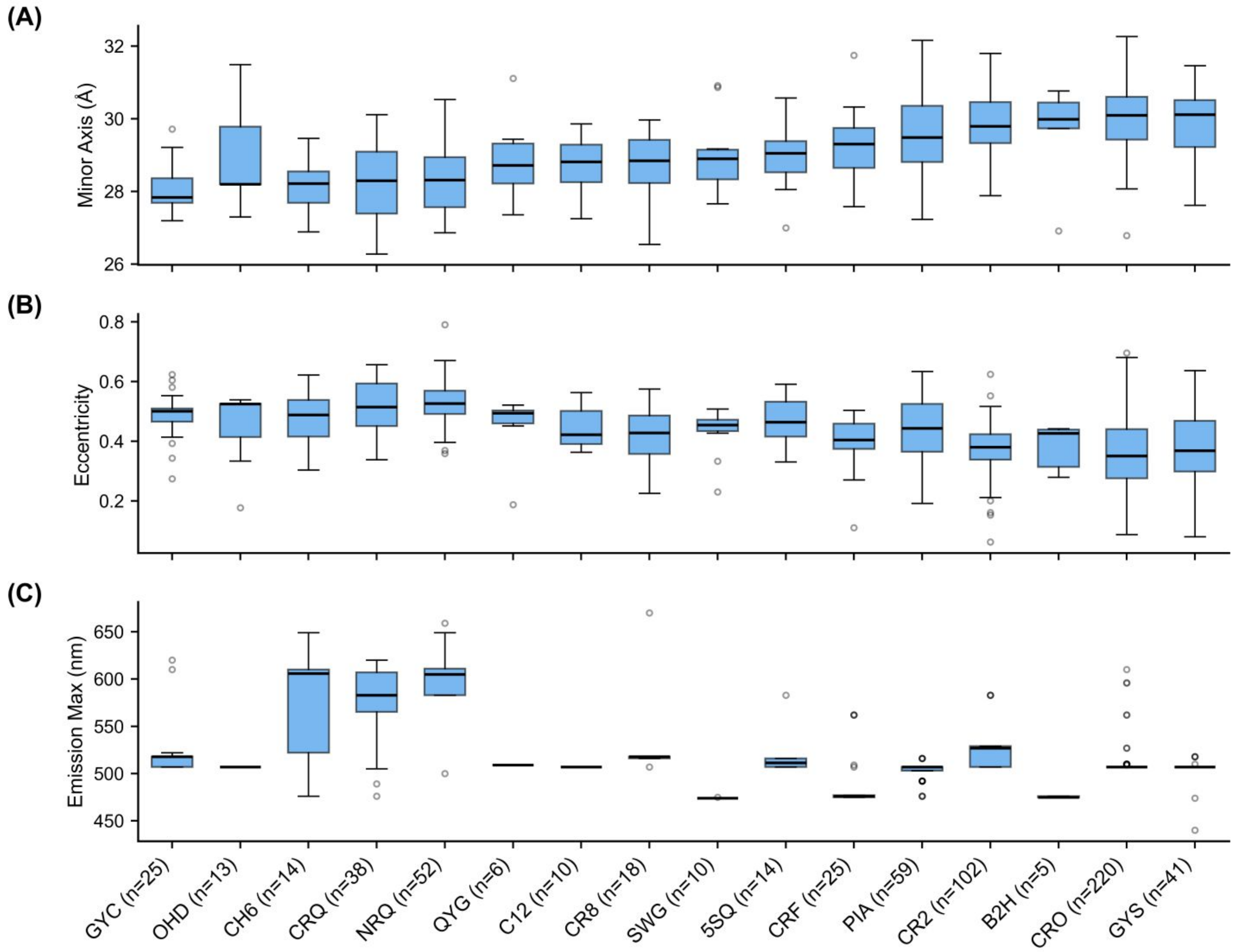

Figure S8. Barrel geometry by chromophore residue type. Chromophore codes with n < 5 are omitted for legibility; this excludes the four His66-derived (blue) codes (IIC, CRG, CSH, XXY; n = 1, 3, 4, and 1 respectively).
